# Practical Use of Ultrasound in Modern Rheumatology—From A to Z

**DOI:** 10.3390/life14091208

**Published:** 2024-09-23

**Authors:** Tanya Sapundzhieva, Lyubomir Sapundzhiev, Anastas Batalov

**Affiliations:** 1 Department of Propedeutics of Internal Diseases, Faculty of Medicine, Medical University of Plovdiv, 4001 Plovdiv, Bulgaria; sapoundjiev@abv.bg (L.S.); abatalov@hotmail.com (A.B.); 2 Rheumatology Department, University Hospital ‘Pulmed’, 4002 Plovdiv, Bulgaria; 3 Rheumatology Clinic, University Hospital ‘Kaspela’, 4000 Plovdiv, Bulgaria

**Keywords:** ultrasound, rheumatology, musculoskeletal, imaging

## Abstract

During the past 20 years, the use of ultrasound (US) in rheumatology has increased tremendously, and has become a valuable tool in rheumatologists’ hands, not only for assessment of musculoskeletal structures like joints and peri-articular tissues, but also for evaluation of nerves, vessels, lungs, and skin, as well as for increasing the accuracy in a number of US-guided aspirations and injections. The US is currently used as the imaging method of choice for establishing an early diagnosis, assessing disease activity, monitoring treatment efficacy, and assessing the remission state of inflammatory joint diseases. It is also used as a complementary tool for the assessment of patients with degenerative joint diseases like osteoarthritis, and in the detection of crystal deposits for establishing the diagnosis of metabolic arthropathies (gout, calcium pyrophosphate deposition disease). The US has an added value in the diagnostic process of polymyalgia rheumatica and giant-cell arteritis, and is currently included in the classification criteria. A novel use of US in the assessment of the skin and lung involvement in connective tissue diseases has the potential to replace more expensive and risky imaging modalities. This narrative review will take a close look at the most recent evidence-based data regarding the use of US in the big spectrum of rheumatic diseases.

## 1. Introduction

The first published data regarding the use of ultrasound (US) in rheumatology were in the late 1990s, when radiologists performed US examination to differentiate thrombophlebitis from a Baker’s cyst [1]. A few years after this report, the US image of synovitis in rheumatoid arthritis was described again by radiologists [2]. Initially, the US was mostly used for assessment of the large joints. With the technological advances and implementation of high-frequency transducers in the rheumatology practice, the number of published studies about the use of US for assessment of small joints has rapidly increased during the past two decades [3]. The increasing use of US in rheumatology is mostly due to the numerous advantages this imaging technique possesses, namely the lack of ionizing radiation, the cost effectivity, the possibility for simultaneous assessment of many structures and for dynamic evaluation of the area of interest, and the fact that it is patient-friendly and enables a more accurate and less risky performance of a number of joint and periarticular diagnostic and therapeutic procedures [3].

The aim of this narrative review is to present in a clear and structured way the recent advances of US in rheumatology practice.

## 2. US in Inflammatory Joint Diseases

### 2.1. US for Establishing a Diagnosis

**Figure 1:**
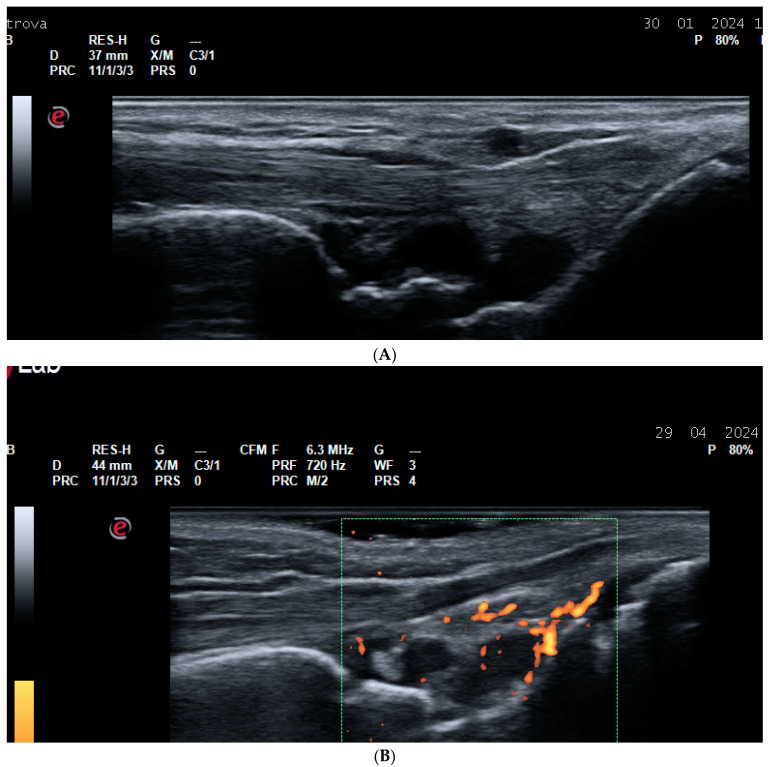

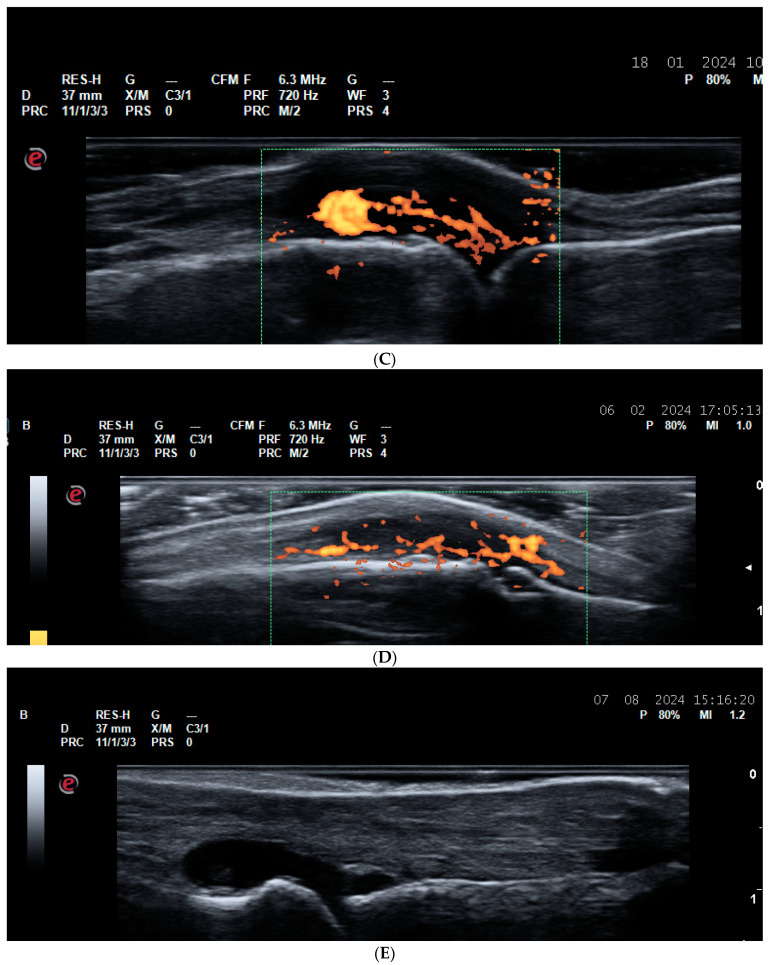
Synovitis. (**A**) A dorsal longitudinal scan of the wrist joint on Gray Scale Ultrasound (GSUS) in an RA patient—Grade 3 synovitis of the radiocarpal and intercarpal joints; (**B**) a dorsal longitudinal scan of the wrist joint on Power Doppler Ultrasound (PDUS) in an RA patient—Grade 2 synovitis; (**C**) a dorsal longitudinal scan of the second metacarpophalangeal (MCP) joint on PDUS in an RA patient—Grade 3 synovitis; (**D**) a dorsal longitudinal scan of the third proximal interphalangeal (PIP) joint on PDUS in an RA patient—Grade 3 synovitis; (**E**) a dorsal longitudinal scan of the first metatarsophalangeal (MTP) joint on GSUS—effusion and grade 3 synovitis.

**Figure 2:**
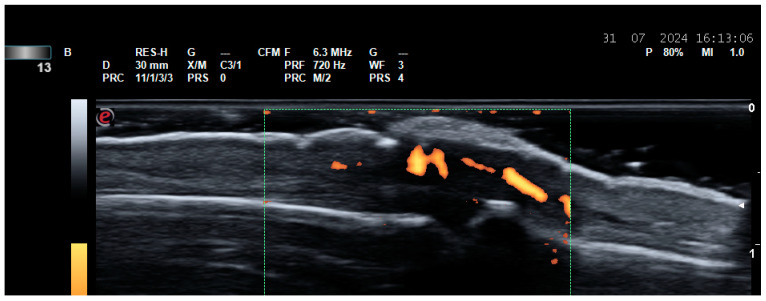
Paratenonitis—inflammation of the finger extensor tendon on PDUS. A dorsal longitudinal scan of the third PIP joint in a PsA patient.

**Figure 3:**
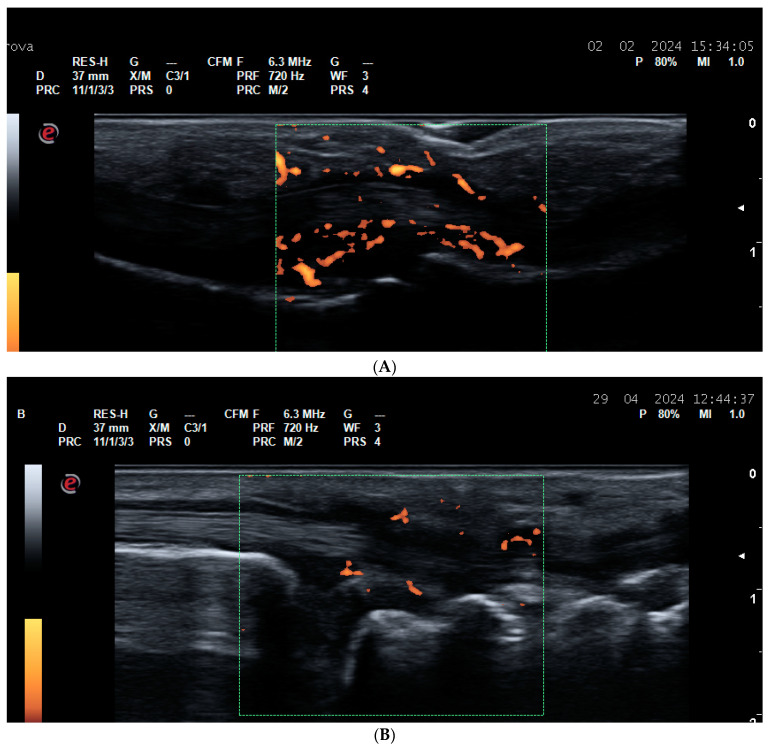

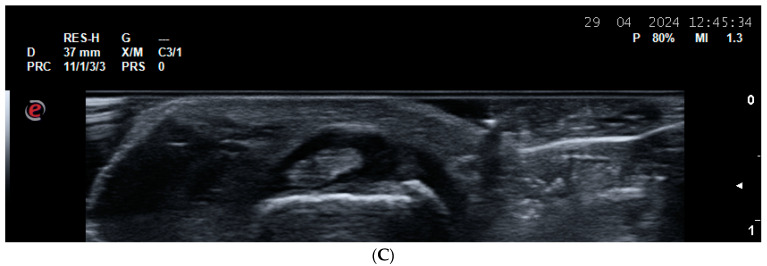
Tenosynovitis. (**A**) Finger flexor tenosynovitis—a palmar longitudinal scan of the second PIP joint in a PsA patient—Grade 3 tenosynovitis of the finger flexor tendon on PDUS; (**B**) tenosynovitis of the VI extensor compartment of the wrist—extensor carpi ulnaris tedon—in an RA patient—ulnar longitudinal scan—grade 2 tenosynovitis on PDUS; (**C**) tenosynovitis of the VI extensor compartment of the wrist—extensor carpi ulnaris in an RA patient—ulnar transverse scan—grade 2 tenosynovitis on PDUS.

**Figure 4:**
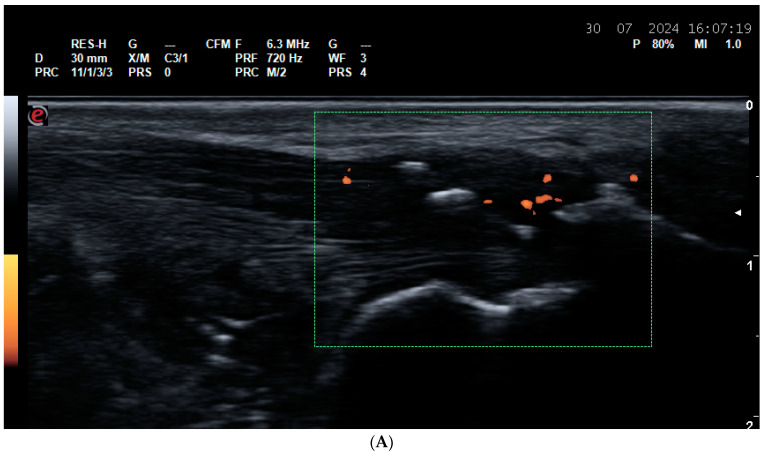

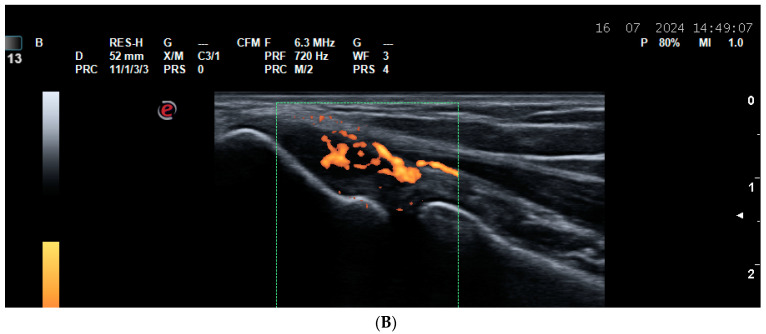
Enthesitis. (**A**) Enthesitis of the enthesis of the Achilles tendon on the calcaneus in a patient with ankylosing spondylitis. A thickened hypoechoic enthesis, with loss of the normal fibrillar pattern, calcifications, enthesophytes, exhibiting a PD signal; (**B**) enthesitis of the common extensor tendon enthesis on the lateral epicondyle of the humerus—thickened and hypoechoic enthesis, loss of the normal fibrillar pattern, exhibiting a PD signal.

The US has been proven to be far more sensitive than the physical examination for the detection of synovitis, tenosynovitis, and enthesitis, thus being helpful in the establishment of early diagnosis of rheumatoid arthritis (RA) and spondyloarthritis (SpA) [4]. Please see Figure 1, Figure 2, Figure 3 and Figure 4. Subclinical synovitis and enthesitis is a common finding in the early stage of inflammatory joint diseases [4]. Therefore, the use of US in patients presenting with a new-onset inflammatory type of joint pain is of crucial importance in order not to delay diagnosis [4]. The number of US-detected inflamed joints and entheses has been proven to be greater than the number of inflamed joints/entheses found during physical examination [5,6,7,8,9,10].

The US-detected subclinical synovitis has even been proven to be a predictor of developing arthritis in patients with arthralgia, and, vice versa, the absence of joint inflammation from the US assessment is a negative predictor [11,12,13,14].

The US has even higher sensitivity than conventional radiography for the detection of structural damage, namely early erosions [15,16], and similarly to US-detected synovitis, US-detected erosions in arthralgia patients are also predictive of the development of arthritis [17,18].

#### 2.1.1. US in RA Diagnosis

The key elementary lesions detected by US in RA are synovitis, tenosynovitis, bursitis, erosions, synovial cysts, and rheumatoid nodules [4]. In patients with early RA, the number of inflamed joints and tendons assessed by US has been proven to be greater than the one assessed by physical examination, meaning that US can visualize subclinical inflammation; thus, patients previously considered to have oligoarthritis may be diagnosed with polyarthritis [4,5,6,19]. In 2019, a US multinational working group published definitions for the main lesions detected by US [20]. It is important to assess the synovial hypertrophy both on gray scale and on Power Doppler (PD) US, the latter providing valuable information for the activity of the synovitis [21].

#### 2.1.2. US in PsA Diagnosis

The inflammation of the entheses, termed enthesitis, along with synovitis, is considered to be the main inflammatory finding in patients with SpA, especially PsA [22,23]. Enthesitis has been documented to affect approximately 35% of PsA patients, being far more prevalent in PsA than in other joint diseases [24]. On the US, enthesitis presents with two types of lesions—inflammatory, consisting of the increased thickness of the enthesis, a hypoehogenic appearance, loss of the normal fibrillar pattern, and a positive PD signal, and structural, defined as the presence of enthesophytes, calcifications, or erosions at the entheseal level, considered to be no more than 2 mm distal to the tendon insertion on the bony surface [20]. According to the literature, the most commonly affected entheses in PsA are plantar fascia and Achilles tendon enthesis on the calcaneus, and the common extensor tendon enthesis on the lateral epicondyle of the humerus [24].

The US reveals subclinical enthesitis, underlying the importance of screening with US the target entheses in PsA in patients with early arthritis [4,9,10].

#### 2.1.3. US Role for the Differential Diagnosis between RA and PsA

The US can be very helpful in the differential diagnosis between arthritides in the early stage, when clinical symptoms and physical findings may be similar, and specific disease-related features are still missing [25,26,27]. For example, the recognition of a specific pattern of inflammation by US can aid the rheumatologist to establish the correct diagnosis. Both RA and psoriatic arthritis (PsA) may present with symmetric involvement of the small joints of the hands. In the absence of specific erythematous papulous-squamous rash and specific antibodies, the early diagnosis can be challenging. The detection of mini-enthesitis by US is very specific for the diagnosis of PsA as compared to RA [25,28]. Mini-enthesitis encompasses several US-detected lesions, for example, inflammation of the paratenon of the finger extensor tendon (paratenonitis), inflammation of the small entheses of the central slip of the extensor tendon at the level of the proximal phalanx, or of the distal insertion at the distal phalanx, inflammation of the collateral ligaments of the small finger joints, inflammation of the pulley of the flexor tendon, and subcutaneous swelling around the finger flexor tendon (the so-called pseudotenosynovitis) [25].

### 2.2. US for Assessment of Disease Activity and Monitoring Response to Therapy

In order to use the ‘treat-to-target’ strategy for the control of inflammation in inflammatory joint diseases, a number of studies recommend to use US to more accurately assess the disease activity state in addition to the clinical disease activity indices [29,30,31,32,33].

The detailed US assessment of 28 joints is time consuming; therefore, a number of studies have been conducted exploring whether a reduced joint count is reliable enough to reflect disease activity and sensitive enough to reflect the therapeutic response. Currently, 7-, 8-, 9-, and 12-joint sets have been proven to correlate with the extensive and time-consuming US assessment of 24 or more joints [34,35,36,37].

In addition to the role of US for assessing response to therapy in RA, a number of studies have used US to detect early treatment efficacy in PsA patients [38,39,40].

Nevertheless, the results of two big randomized trials (the TASER and the ARCTIC) exploring the added value of US assessment of disease activity as compared to the classical assessment by composite clinical indices have revealed non-superiority of the imaging versus clinical evaluation for the treatment outcomes, thus highlighting the necessity for future studies to prove that US-based decision-making regarding management of patents with RA offers more benefits for the patient outcomes than clinical examination alone [41,42].

### 2.3. US for Assessment of the State of True Remission

The purpose of treating RA is to reach remission because it is associated with the best functional and structural outcomes for the patients [43]. According to the latest European League Against Rheumatism (EULAR) recommendations for management of RA, rheumatologists should define remission based on two definitions—the Boolean or index-based American College of Rheumatology (ACR)-EULAR remission definition [44]. Nevertheless, a number of RA patients still experience radiographic progression despite being in a state of remission [45]. A possible explanation for this concordance is the fact that many patients in clinical remission have evidence of persistent PD positive synovitis seen on US [37,43,46,47]. The practical implication for defining which patients are in the state of deep remission (clinical and US) is the fact that the risk of a disease relapse after drug tapering is the smallest [48,49,50]. Thus, the US may help the rheumatologist to decide which of the patients in clinical remission are suitable for attempting to decrease the dose or increase the interval for biologic drug administration [49,50]. A systematic review and meta-analysis have proven that subclinical joint inflammation, detected by US, is a predictor for a future flare and structural progression in RA patients in remission according to the composite disease activity indices [51].

After having discussed the role of US for the assessment of remission, the benefit of incorporating US in the management of patients with difficult-to-treat RA (D2T-RA) has also been proven [52]. In a recent study by David et al., US was used to distinguish different phenotypes of D2T RA patients—an inflammatory, characterized by US evidence of synovitis or tenosynovitis, versus a non-inflammatory [52]. The study shows that as many as 43% of D2T-RA do not to have evidence of joint or tendon inflammation and have higher prevalence of a high body mass index and fibromyalgia [52]. This finding has a practical implication; those patients would not benefit from escalation of therapy or switching to another drug class, as compared to the patients with the inflammatory phenotype, suggesting that in this clinical scenario, US-driven patient management may prove to be superior versus a clinically-driven strategy [52,53].

## 3. US in Degenerative and Crystal-Related Joint Diseases

### 3.1. US Use in Patients with Osteoarthritis (OA)

**Figure 5:**
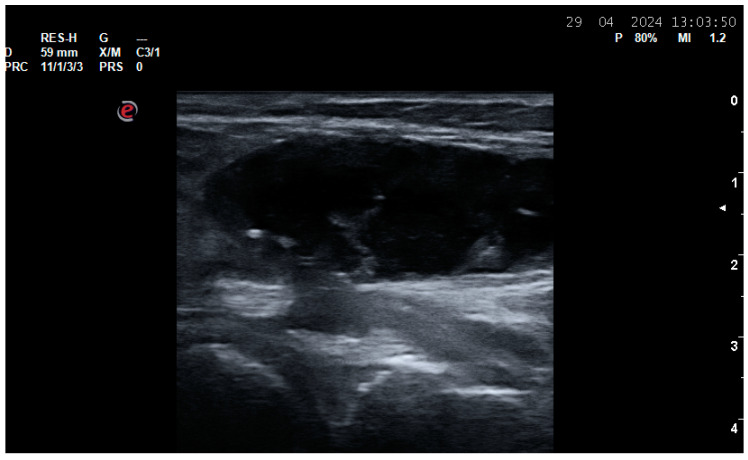
A Baker’s cyst (semimembranosus-gastrocnemius bursa) of the knee joint—a posterior longitudinal scan of the medial tibio-femoral joint space shows a cyst with a heterogenous predominantly anechoic internal structure.

The first recommendations describing the role of different imaging modalities for OA were published in 2017 [54]. For assessment of soft tissue pathology, accompanying OA, and guiding needle placement on difficult to access joints, joints with severe deformity, or in obesity patients, US and MRI are the imaging methods of choice [54]. A well-known discrepancy between the level of pain and the radiographic changes exists in OA [54]. In addition to the physical examination and radiography of the joints, US can detect the source of pain for the individual patients with OA, thus improving patient management. A number of joint and peri-articular soft-tissue pathologies can be easily detected by US, the most common for the knee OA being intra-articular effusion, pes anserinus bursitis, enthesopathy of the proximal and distal patellar tendon and of the quadriceps tendon, Baker’s cyst, and iliotibial band tendinitis, all of which can be easily relieved by a local corticosteroid injection [55]. Please see Figure 5. Moreover, in comparison to radiographic changes that do not correlate with patients’ pain, US-detected lesions have been found to correlate to a great extent with pain intensity, as measured on a visual analogue scale (VAS) [55].

### 3.2. US in Crystal-Related Arthropathies

#### 3.2.1. Gout

**Figure 6:**
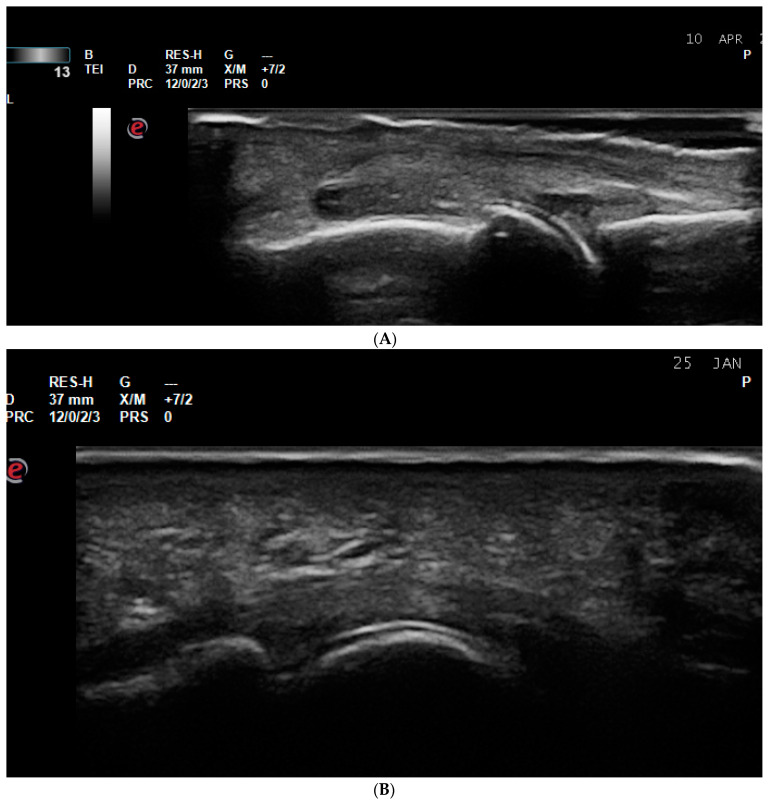
Double-contour sign (DCS) in a gout patient. (**A**) A dorsal longitudinal scan of the second MCP joint in a gout patient. Grade 3 synovitis and DCS of the hyaline cartilage of the metacarpal head; (**B**) a plantar longitudinal scan of the second MTP joint in a gout patient. DCS of the hyaline cartilage of the metatarsal head.

The detection of the cartilage double-contour sign (DCS), reflecting the deposition of uric acid crystals over the hyaline cartilage, is included in the 2015 classification criteria for gout [56]. Please refer to Figure 6. The proven high specificity of the US lesions detected in gout (DCS; snowstorm appearance of the joint effusion, tophus, bony erosions) makes it a valuable diagnostic tool [57,58]. The joints and tendons that frequently show US evidence of urate crystals deposition, with the best balance between sensitivity and specificity, are the first metatarsophalangeal, the tibio-talar, the second metacarpophalangeal and the knee joint, and the patellar and triceps tendons [57].

In addition to its role in the establishment of the diagnosis of gout, a number of studies have applied US for monitoring treatment response during urate-lowering therapies [59,60]. Recently, the value of US as a predictive imaging tool for gout flares has been established in two studies [61,62].

#### 3.2.2. Calcium Pyrophosphate Deposition Disease (CPPD)

**Figure 7:**
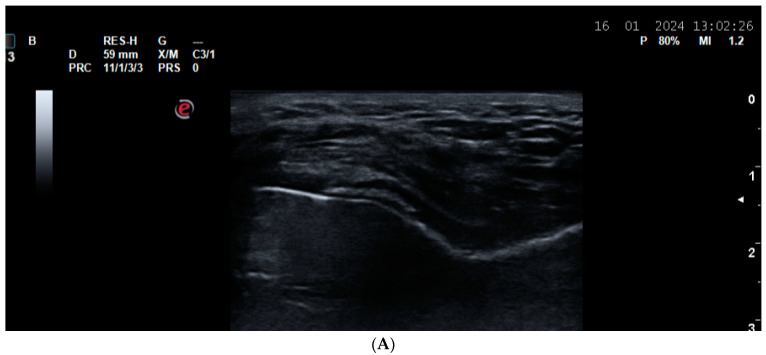

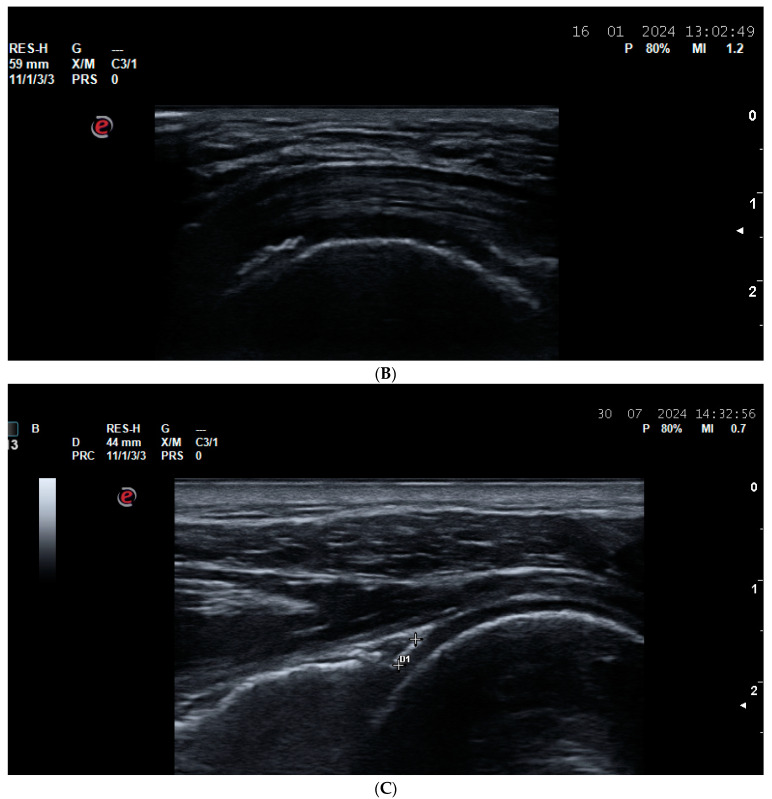
Calcium pyrophosphate deposition disease (CPPD). (**A**) A posterior longitudinal scan of the tibio-femoral joint in a CPPD patient. A linear hyperechoic calcification within the femoral hyaline cartilage. (**B**) A posterior transverse scan of the tibio-femoral joint in a CPPD patient. A linear hyperechoic calcification within the femoral hyaline cartilage. (**C**) A posterior scan of the gleno-humeral joint in a CPPD patient. A hyperechoic calcification (between the cursors) in the fibrocartilage of the labrum.

The reliability of US in the assessment of the triangular fibrocartilage of the wrist in CPPD using conventional radiography as a reference method has been proven [63,64]. Musculoskeletal Ultrasound (MSUS) has been proven to be more sensitive than conventional radiography in identifying CPP crystals [65]. In 2017, Filippou et al. developed definitions for elementary lesions typical for CPPD in the hyaline cartilage and fibrocartilage of the knee joint [66]. A year later, the same working group established the reliability of US-detected CPP deposits for other locations—the triangular fibrocartilage of the wrist joint and acromio-clavicular joint [67]. Please see Figure 7. The new 2023 Classification Criteria for CPPD emphasize the important role of US evidence of CPP crystal deposition, due to the fact that the highest number of points of the two imaging domains comprise almost 50% of the weighting [68]. A semi-quantitative scoring system for US-detected CPPD burden was developed in 2023, which opens the way for US monitoring of treatment response in trials evaluating new treatment options for CPPD [69].

The US can even help in the differential diagnosis between gout and CPPD (pseudogout), because uric acid crystals deposit on the surface (creating the DCS), while the CPP crystals are located in the middle layer of the hyaline cartilage [70]. Recently, the pseudo-double contour sign has been proven to be a hallmark for CPPD—CPP crystals can deposit within the ligament located above the hyaline cartilage, thus mimicking uric acid crystals [70]. Dynamic scanning may be used to differentiate between the DCS in gout and pseudo DCS in CPPD—the DCS moves together with the hyaline cartilage during joint movement, whereas the pseudo DCS and the cartilage move in the opposite direction [70].

## 4. US in Systemic Connective Tissue Diseases

### 4.1. Systemic Sclerosis (SSc)

The US has various applications in the assessment of patients with SSc, namely for evaluation of skin fibrosis, lung involvement, joint and peri-articular pathology, calcifications, and digital tip ulcers [71].

Skin US—The modified Rodnan Skin Score (mRSS) is the most widely accepted method for assessment of the skin fibrosis in SSc, although it possesses a lower reproducibility and can miss subtle skin changes. The US can detect subclinical dermal thickening, has a higher intra- and inter-observer reproducibility, and is more sensitive to change during treatment [72,73,74]. During the last decade, the introduction of very high frequency probes (more than 18 MHZ) and new variations of the classic B-mode US assessment (classical and shear wave elastography) has brought skin US to a new level [73,74]. The World Scleroderma Foundation (WSF) skin US group has even issued a set of recommendations regarding the way of performing and reporting a skin US [74].

US of digital tip ulcers (DUs)—In the past, inspection of the DUs was the only way to describe their characteristics—size, depth, presence of calcifications or infection—but the introduction of US has shed a new light on the topic and has provided objectivity for estimation of size, depth, and even response to treatment. Until now, US for the assessment of DUs has been partially validated and future research for proving its construct validity and reliability is still warranted [75].

US of joints, enthesis, tendons—Joint pain in patients with SSc may be the result of a great number of underlying pathologies, for example, joint inflammation, tendon or enthesis inflammation, calcinosis, acro-osteolysis, nerve entrapment, or deposition of calcifications, all of which can be easily assessed by US [76]. Joint synovitis and tendon friction rubs (TFRs) are independent predictive factors for disease progression in patients with early SSc. With the help of US, the tenosynovitis type may be determined—an inflammatory (anechoic or isoechoic tendon sheath widening) versus sclerosing (hyperechoic thickening of the tendon sheath), the latter being very specific to SSc patients. In addition, the extensor tendons tend to be more frequently affected in SSc patients as compared to the flexor tendons [76,77].

The presence of synovitis and sclerosing tenosynovitis (non-inflammatory, the reason for the TFRs) has been established as a risk factor for a progressive disease [78].

Lung US—Interstitial lung disease (ILD) is the major cause of death in SSc patients, necessitating early detection to improve the outcomes [79]. Currently, the golden standard for evaluation of ILD is high-resolution computed tomography (CT) [80]. The radiation exposure and high cost are disadvantages of this imaging modality, which is why research is focused on new imaging methods that may detect the ILD early [80]. Lung US has been proven to correlate with CT to a great extent [81,82]. The findings that are typical of ILD include the presence of B-lines and pleural line alterations [81,82]. During the last years, many researchers have described different scanning protocols for lung US, including a different number of intercostal spaces—10, 14, 58, and 72—but until now, neither of them have been accepted, and research is still ongoing to find which is the number of intercostal spaces with the best balance between sensitivity and time needed for assessment [83,84,85,86,87]. A meta-analysis by Radić et al., published in 2023, revealed that lung US has high sensitivity for the detection of ILD [88]. Another meta-analysis has proven the 14-intercostal space US protocol to be sensitive enough, as compared to the more extensive US protocols, assessing a greater number of intercostal spaces [89].

### 4.2. Systemic Lupus Erythematosus (SLE)

Musculoskeletal complaints are extremely common in lupus patients [90]. The disease activity indices, commonly applied in clinical practice, consider the musculoskeletal domain active only if there is presence of swollen joints during physical examination [91]. In SLE patients, subclinical joint and tendon inflammation, detected by US, is frequently reported [92]. For example, Zayat et al. detected the presence of synovitis and tenosynovitis in 27% of patients with no clinical evidence of joint or tendon inflammation [93].

In addition to the joint and tendon inflammation, entheses inflammation, detected by US, has also been found in a great percentage of lupus patients—29.2 to 71%—and has been proven to correlate with disease activity [94,95,96]. It has even been suggested that a distinct phenotype of SLE, presenting with enthesitis, more joint synovitis, less kidney involvement, and an insufficient response to treatment with anti-CD20 drugs, may be a step toward a personalized patient management in SLE [95].

The clinical significance of MSUS in SLE patients would be to improve therapeutic management. In a study by Mahmoud et al., corticosteroid treatment had led to improvement in clinical and patient-reported outcomes to a greater extent if synovitis had been detected by US [97].

### 4.3. Inflammatory and Non-Inflammatory Myopathies and Sarcopenia

The US of the skeletal muscles may be helpful in the diagnosis of inflammatory myopathies, with findings being different in relation to the phase of the disease—in the acute stage of myositis, the oedema in the striate muscle leads to an increase in the thickness and a more hypoechoic appearance, whereas in the chronic stage, the atrophy of the muscle and replacement of the muscle fibers with fat tissue leads to a more hyperechoic appearance as compared to a healthy muscle [98]. The US assessment of the muscles and application of a scoring system for muscle echogenicity have been proven to possess a high intra- and inter-observer reliability [99]. A systematic review by the OMERACT found different outcome US measures in myositis, including echogenicity (used in most of the published studies), muscle thickness, perimysial septal count, fascial thickness, and vascularity [100]. Strain and shear wave elastography were also used in a number of studies with variable results reported by the authors [101,102,103]. A few longitudinal studies use US to monitor the treatment response [104,105]. At the current moment, muscle US has demonstrated construct validity, but further research is warranted to prove its reliability, discriminatory ability, and to validate quantitative scoring systems for US assessment of the patients with inflammatory myopathies [99,105].

In addition to the US’s role in assessing patients with inflammatory myopathies, recent research focuses on the utility of the imaging modality to evaluate the muscle volume, thus being useful as a screening tool for sarcopenia, a common finding in patients with inflammatory joint diseases, such as RA, which has an impact on a number of clinical outcomes, for various chronic diseases [106,107,108].

Besides the role of US in the diagnostic work-up of inflammatory myopathies, a number of non-inflammatory muscle diseases, including neuromuscular pathology, have been an area of research focusing on the potential utility of US in two domains: (1) screening for congenital and acquired neuro-muscular diseases (namely Pompe disease, spinal muscular atrophy, Duchenne muscular dystrophy, congenital myotonias, amyotrophic lateral sclerosis, etc.); (2) for monitoring disease course and treatment efficacy in both [109,110,111,112].

### 4.4. US in Primary Sjogren Syndrome (pSS)

**Figure 8:**
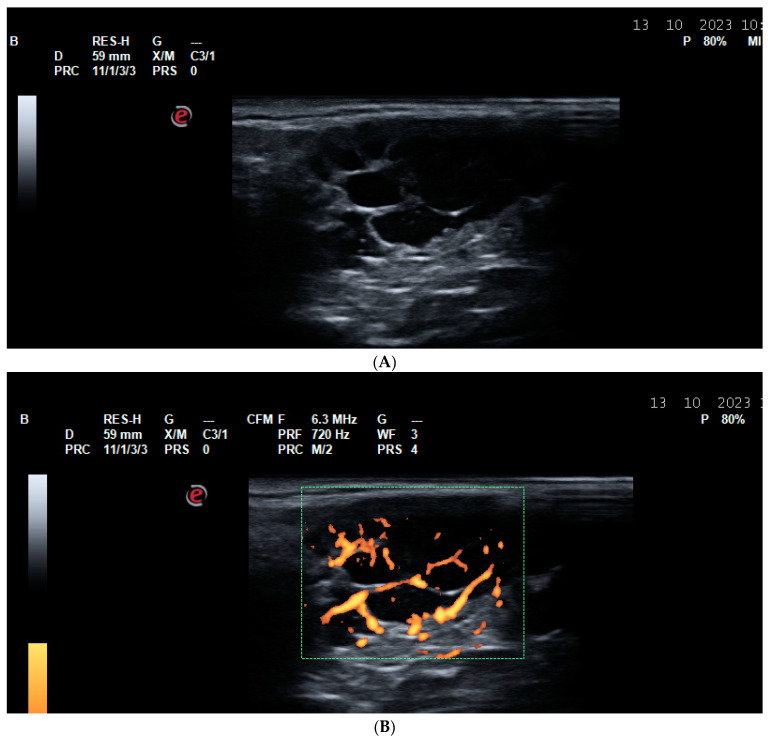
Lymphoma of the parotid gland in a patient with Sjogren Syndrome. (**A**) Parotid gland lymphoma on GSUS—a mass with a heterogenous internal structure, a lobulated appearance, and irregular, poorly defined borders; (**B**) parotid gland lymphoma on PDUS.

The US of the major salivary glands is currently the imaging modality of choice in patients with primary Sjogren syndrome (pSS) [113]. The semi-quantitative US score, developed by the OMERACT, has been proven to possess very high inter- and intra-observer reliability, regardless of the years of experience with US of the performing physician [114,115]. Furthermore, when US of the major salivary glands is included in the classification criteria, their sensitivity has been found to increase [116,117]. In addition to the establishment of a diagnosis of pSS, US of the salivary glands may be used to differentiate pSS from other conditions that affect the parotid and submandibular glands. Two US patterns may be defined: a diffuse and a focal one. In addition to pSS, IgG4-related disease, amyloidosis, sarcoidosis, hepatitis ‘’C’’ and HIV infections, and prior radiation exposure of the head and neck may lead to a diffuse US pattern of inhomogenuity of the major salivary glands [118,119,120]. A focal US pattern of the parotid or submandibular gland should raise the suspicion for a neoplastic lesion, namely lymphoma, considering that pSS is the rheumatic disease with the highest risk for the development of lymphoproliferative disorders, especially non-Hodgkin B-cell lymphoma (NHL), of which mucosa-associated lymphoid tissue-NHL is the most common one [121,122]. A grade of 3 according to the OMERACT scoring of the major salivary glands and several suspicious for lymphoma US characteristics of the focal lesions have been proven to be alarming for a transition to a lymphoma and the need to perform a core-needle biopsy (CNB): an oval shape, well-demarcated margins, very hypoechoic echotexture, inner septa, increased vascularization with an intense color Doppler signal, and the presence of posterior acoustic enhancement [123]. Please see Figure 8. Besides lymphoma, US can visualize other focal lesions in the parotid and submandibular glands, for example, Warthin tumors and pleomorphic adenomas [124].

The US can guide CNB of the salivary glands, during which a tissue sample is obtained, enabling immunohistochemical staining and performing of flow cytometry, two methods that are crucial in case there is a suspicion of a lymphoma [125,126]. CNB is considered a safe procedure with a minimal risk of complications, namely facial nerve damage, as compared to the open biopsy, and at the same time, possesses the same diagnostic accuracy [126].

The US of the salivary glands in pSS may be used as an imaging biomarker to monitor disease activity and the response to therapy [127,128]. A few published studies evaluate the efficacy of biologic treatment for pSS by assessing the change of a US score of the major salivary glands [127,128].

In addition to the classical US, elastography (strain, shear wave, and acoustic radiation force impulse—ARFI) has been recently investigated as an imaging tool that can complement the standard US assessment of the salivary glands to diagnose more accurately pSS by increasing the sensitivity and specificity of the method [129,130]. An observational study, performed by Bădărînză et al., found that the higher stiffness of the NHL, assessed by shear wave elastography, can provide an added diagnostic value to the standard US of the salivary glands when there is a suspicion for NHL [131].

## 5. US in Polymyalgia Rheumatica and Systemic Vasculitides

### 5.1. Polymyalgia Rheumatica (PMR)

The valuable role of US in polymyalgia rheumatica (PMR) is highlighted by its inclusion in the 2012 ACR/EULAR classification criteria [132]. A systematic review points out that subacromial-subdeltoid bursitis is the most helpful US lesion for the establishment of the diagnosis of PMR. Other typical US findings in PMR include tenosynovitis of the long head of the biceps tendon, glenohumeral synovitis, trochanteric bursitis, and hip joint synovitis [133].

Another interesting pathological finding common in PMR patients that can be easily detected by US is cervical or lumbar interspinous bursitis [134,135]. An interesting study, conducted by Conticini et al., revealed that baseline US findings in PMR may predict a change in the diagnosis after 12 months [136]. The baseline US lesions that were most predictive of persistence of the initial PMR diagnosis were bilateral subacromial-subdeltoid (SASD) bursitis and biceps tenosynovitis [137].

According to the EULAR recommendations for management of PMR patients, before the initiation of corticosteroid treatment, an alternative diagnosis, mimicking PMR, should be excluded [137]. The US can be extremely helpful in the differential diagnosis [138]. Considering that elderly-onset RA is the first diagnosis to exclude, and what is known that RA presents mostly with synovial hypertrophy, whereas PMR presents with predominant effusion, a study reveals that US evidence of a high-grade (second or third in a semi-quantitative score from 0 to 3) proliferative SASD is suggestive of elderly-onset RA [139]. In another study, the US evaluation of the acromio-clavicular joint has been proven to be helpful in the differential diagnosis of PMR in patients presenting with polymyalgic syndrome, because synovitis of this joint, together with the detection of intra-articular hyperechoic deposits, has helped to establish a diagnosis of CPPD [140].

### 5.2. Vasculitides

The US is helpful in the assessment of large-vessel vasculitides, namely giant-cell arteritis (GCA) and Takayasu arteritis (TAK) [141]. The US signs of vascular inflammation are part of the imaging domain in the 2022 ACR/EULAR classification criteria for GCA and TAK, and in GCA, US evidence of vasculitis adds five points, having the same weight as histology [142,143].

Furthermore, the latest EULAR recommendations, recently published, highlight the crucial role of US in the diagnostic algorithm of GCA and TAK, stating that US assessment of the temporal and axillary arteries is the first imaging method that should be used in the former, and an alternative to MRI in the latter [144]. Thanks to the growing body of evidence, US replaces the golden standard for diagnosis of GCA, namely temporal artery biopsy [120]. In a patient, in whom there is suspicion of GCA, the arteries that need to be scanned by US are the right and left common superficial temporal artery with its two branches (the parietal and the frontal) and the axillary artery [145].

When performing vascular US, it is very important to consider the potential pitfalls, namely the following: (1) a non-sufficient Doppler signal in case the Doppler window is not angulated; (2) an inappropriately high gain, leading to underestimation of the ‘halo sign’; (3) gain is too low, leading to the ‘pseudohalo sign’; (4) atherosclerosis, having impact on the measurement of intima–media thickness, is quite a common pitfall, considering the age of the patients with suspected GCA. The plaques that form atherosclerosis are rather hyperechoic, asymmetric, and rarely affect the temporal arteries, but still should not be overlooked [146].

An important role of US is to screen for vascular inflammation in PMR patients. More than a fifth of PMR patients have a positive halo sign, meaning that they have a subclinical GCA [147]. The clinical implication of this data is that a PMR patient showing signs of GCA from the US assessment may need to be treated as if he has a classical GCA, meaning there is a need to start with a higher corticosteroid dosage [147]. In line with that, even though there are still not enough data to recommend that all PMR patients need to have a vascular US to detect subclinical GCA, some preliminary data suggest that rheumatologists should be educated to perform vascular US to better manage PMR patients [147].

In addition to the role of US for the diagnosis of vasculitides, some evidence exists suggesting that it may be a tool to monitor disease activity and response to therapy, because intima–media thickness correlates with disease activity [148,149]. The OMERACT group has created a global score, based on the IMT values, that has been proven to be reliable [148]. Nielsen at al. have tested the sensitivity to change of different US vascular scores and have found that all of them can be used to monitor treatment efficacy, highlighting one of the scores as being a potential outcome measure in trials evaluating the efficacy of different drugs [149].

## 6. US for Assessment of Peripheral Nerves

The specific pattern of nerve appearance on a transverse scan—fascicular (honey-comb like appearance)—as compared to tendon appearance, which has a fibrillar pattern, in addition to the fact that tendons exhibit anisotropy when the US beam is not perpendicular to the tendon fibers, contributes to the US’s ability to easily differentiate a tendon from a nerve [150].

The US is able to visualize both the changes in the appearance of the compressed peripheral nerve, to provide information regarding the etiology of the entrapment, as well as to guide the needle placement in a number of therapeutic procedures [151]. The US has been proven to be extremely useful in the diagnostic assessment of entrapment syndromes, the most common of which for the upper limb being the median nerve in the carpal tunnel and the ulnar nerve in the Guyon’s canal, and for the lower limb, the posterior tibial nerve entrapment in the tarsal tunnel and the common peroneal nerve as it courses near the neck of the fibula [152]. The US criteria for nerve entrapment have already been published [153,154].

## 7. US for Guiding the Needle in Invasive Procedures

As compared to blind injections, US-guided invasive procedures in rheumatology have been proven to pose greater accuracy, efficacy, and safety [155]. According to the EULAR recommendations for intra-articular therapies, US may be used to guide needle placement to improve the accuracy of the procedure [156]. The European Society of Musculoskeletal Radiology (ESSR) has outlined the clinical indications for the image-guided (with US having a central role) invasive procedures for each of the joints of the upper and lower limb, as well as for the peripheral nerves [157,158,159,160,161,162].

A systematic review found that US-guided gleno-humeral joint injection was accurate in 92.5% of cases versus 72.5% for the blind injection [163]. Regarding the lower limb joints, a study has found that the accuracy of blinded hip joint injections depends on the radiological grade of the hip osteoarthritis (OA). In grade II, according to Kellgren–Lawrence grading, the reported accuracy of blind hip joint injections is 74%, dropping to 61.3% in grade III hip OA [164]. Regarding the knee, the accuracy of the blind injections depends on the approach—from 77.3% in the midpatellar approach to 95.74% in the superolateral approach, as compared to the US-guided knee joint injections, being successful in more than 95% of cases [165].

Nevertheless, the majority of published studies prove the greater accuracy of US-guided versus blind injections in articular and periarticular pathology, and a systematic analysis failed to prove that the former are superior in terms of patient outcomes [166,167,168].

There is a growing body of evidence not only for the role of US in joint procedures, but also for fascia hydrorelease, and injection of a number of peri-articular structures, including entheses, bursae, tendon sheaths, and nerves [169,170].

## 8. Artificial Intelligence in Rheumatology

During the past 10 years, artificial intelligence (AI)-based assessment of US lesions and computer-aided diagnosis have been extensively studied in order to overcome the limitations of MSUS imaging, namely reliability and inter-observer variability.

One of the potential implementations of AI in MSUS is for scoring synovitis, thus reducing the inter- and intra-observer variability. The most important barrier to overcome, however, is to discriminate the synovial hypertrophy from the peri-articular tissue, including bony landmarks, and skin layers, in cases of heterogenous echogenicity [171]. Deep learning (DL) has been used for synovitis grading by utilizing a connectivity algorithm for the segmentation of the bone region [172]. Neural network technology has been proven to be helpful in the assessment of disease activity on PD US images according to the OMERACT-EULAR Synovitis Scoring system [173].

DL-based segmentation has been successfully used to find the location of a number of peripheral nerves (median, radial, ulnar, etc.) to assist in performing various nerve blocks [174,175].

AI has even been used to determine cartilage thickness in knee osteoarthritis [176].

The growing body of evidence regarding DL and AI use in US imaging in rheumatology will become the basis for decreasing the variability and increasing the reliability of MSUS [177].

## 9. Conclusions

The technological advances, the increasing availability of high-end US machines, and the growing number of organized US courses for continuing the education of rheumatologists have contributed to the growing body of evidence regarding the utility of US in daily rheumatology practice, outlining the advantages of the imaging modality in the management of patients with all types of rheumatic diseases—from the inflammatory arthropathies, degenerative and metabolic joint diseases, to the connective tissue diseases and systemic vasculitides. Now, the US is being extensively used in rheumatology not only for assessment of joints and peri-articular tissues, but also for evaluation of the skin, lung, vessels, muscles, and nerves, both for diagnostic purposes and for guiding needle placement in a number of invasive procedures. The recent advances of AI in US imaging will open new horizons in the field of US in the near future.

## Data Availability

Not applicable.

## References

[1] McDonald D.G., Leopold G.R. (1972). Ultrasound B-scanning in the differentiation of Baker’s cyst and thrombophlebitis. Br. J. Radiol..

[2] Cooperberg P.L., Tsang I., Truelove L., Knickerbocker W.J. (1978). Gray scale ultrasound in the evaluation of rheumatoid arthritis of the knee. Radiology.

[3] Sapundzhieva T.L., Karalilova R., Batalov A. (2020). Musculoskeletal Ultrasound in Rheumatology—New Horizons. Folia Medica.

[4] Kaeley G.S., Bakewell C., Deodhar A. (2020). The importance of ultrasound in identifying and differentiating patients with early inflammatory arthritis: A narrative review. Arthritis Res. Ther..

[5] Saku A., Furuta S., Kato M., Furuya H., Suzuki K., Fukuta M., Suehiro K., Makita S., Tamachi T., Ikeda K. (2020). Experience of musculoskeletal ultrasound scanning improves physicians’. physical examination skills in assessment of synovitis. Clin. Rheumatol..

[6] Mandl P., Studenic P., Supp G., Durechova M., Haider S., Lehner M., Stamm T., Smolen J.S., Aletaha D. (2020). Doubtful swelling on clinical examination reflects synovitis in rheumatoid arthritis. Ther. Adv. Musculoskelet. Dis..

[7] Quintana-López G., Maldonado-Cañón K., Flórez-Suárez J.B., Méndez-Patarroyo P., Coral-Alvarado P., Calvo E. (2021). Correlation and agreement between physical and ultrasound examination after a training session dedicated to the standardization of synovitis assessment in rheumatoid arthritis patients. Adv. Rheumatol..

[8] Hammer H.B., Kvien T.K., Terslev L. (2017). Ultrasound of the hand is sufficient to detect subclinical inflammation in rheumatoid arthritis remission: A post hoc longitudinal study. Arthritis Res. Ther..

[9] Elnady B., El Shaarawy N.K., Dawoud N.M., Elkhouly T., Desouky D.E.-S., ElShafey E.N., El Husseiny M.S., Rasker J.J. (2019). Subclinical synovitis and enthesitis in psoriasis patients and controls by ultrasonography in Saudi Arabia; incidence of psoriatic arthritis during two years. Clin. Rheumatol..

[10] Macchioni P., Salvarani C., Possemato N., Gutierrez M., Grassi W., Gasparini S., Perricone C., Perrotta F.M., Grembiale R.D., Bruno C. (2019). Ultrasonographic and Clinical Assessment of Peripheral Enthesitis in Patients with Psoriatic Arthritis, Psoriasis, and Fibromyalgia Syndrome: The ULISSE Study. J. Rheumatol..

[11] Di Matteo A., Duquenne L., Cipolletta E., Nam J.L., Garcia-Montoya L., Wakefield R.J., Mahler M., Mankia K., Emery P. (2022). Ultrasound subclinical synovitis in anti-CCP-positive at-risk individuals with musculoskeletal symptoms: An important and predictable stage in the rheumatoid arthritis continuum. Rheumatology.

[12] Garcia-Montoya L., Kang J., Duquenne L., Di Matteo A., Nam J.L., Harnden K., Chowdhury R., Mankia K., Emery P. (2024). Factors associated with resolution of ultrasound subclinical synovitis in anti-CCP-positive individuals with musculoskeletal symptoms: A UK prospective cohort study. Lancet Rheumatol..

[13] Rogier C., Frazzei G., Kortekaas M.C., Verstappen M., Ohrndorf S., van Mulligen E., van Vollenhoven R.F., van Schaardenburg D., de Jong P.H., van der Helm-van Mil A.H. (2022). An ultrasound negative for subclinical synovitis in arthralgia patients: Is it helpful in identifying those not developing arthritis?. Rheumatology.

[14] Collada J.M., Gloria K.L., Castrejón I., Nieto-González J.C., Rivera J., Montero F., González C., Álvaro-Gracia J.M. (2021). Ultrasound in clinically suspect arthralgia: The role of power Doppler to predict rheumatoid arthritis development. Arthritis Res. Ther..

[15] Lopez-Ben R., Bernreuter W.K., Moreland L.W., Alarcon G.S. (2004). Ultrasound detection of bone erosions in rheumatoid arthritis: A comparison to routine radiographs of the hands and feet. Skelet. Radiol..

[16] Jindal G., Bansal S., Gupta N., Singh S.K., Gahukar S., Kumar A. (2021). Comparison of Ultrasonography and X-rays for the Diagnosis of Synovitis and Bony Erosions in Small Joints of Hands in Early Rheumatoid Arthritis: A Prospective Study. Maedica.

[17] Ziegelasch M., Eloff E., Hammer H.B., Cedergren J., Martinsson K., Reckner Å., Skogh T., Magnusson M., Kastbom A. (2021). Bone Erosions Detected by Ultrasound Are Prognostic for Clinical Arthritis Development in Patients With ACPA and Musculoskeletal Pain. Front. Med..

[18] Di Matteo A., Mankia K., Duquenne L., Cipolletta E., Wakefield R.J., Garcia-Montoya L., Nam J.L., Emery P. (2020). Ultrasound erosions in the feet best predict progression to inflammatory arthritis in anti-CCP positive at-risk individuals without clinical synovitis. Ann. Rheum. Dis..

[19] Wakefield R.J., Green M.J., Marzo-Ortega H., Conaghan P.G., Gibbon W.W., McGonagle D., Proudman S., Emery P. (2004). Should oligoarthritis be reclassified? Ultrasound reveals a high prevalence of subclinical disease. Ann. Rheum. Dis..

[20] Bruyn G.A., Iagnocco A., Naredo E., Balint P.V., Gutierrez M., Hammer H.B., Collado P., Filippou G., Schmidt W.A., Jousse-Joulin S. (2019). OMERACT Ultrasound Working Group. OMERACT Definitions for Ultrasonographic Pathologies and Elementary Lesions of Rheumatic Disorders 15 Years on. J. Rheumatol..

[21] Bhasin S., Cheung P.P. (2015). The Role of Power Doppler Ultrasonography as Disease Activity Marker in Rheumatoid Arthritis. Dis. Markers.

[22] Huayong Z., Jun L., Junlan Q., Fan W., Lingyun S. (2017). Ultrasonographic evaluation of enthesitis in patients with ankylosing spondylitis. J. Biomed. Res..

[23] Smerilli G., Di Matteo A., Cipolletta E., Grassi W., Filippucci E. (2021). Enthesitis in Psoriatic Arthritis, the Sonographic Perspective. Curr. Rheumatol. Rep..

[24] Polachek A., Li S., Chandran V., Gladman D.D. (2017). Clinical Enthesitis in a Prospective Longitudinal Psoriatic Arthritis Cohort: Incidence, Prevalence, Characteristics, and Outcome. Arthritis Care Res..

[25] Sapundzhieva T., Karalilova R., Batalov A. (2020). Hand ultrasound patterns in rheumatoid and psoriatic arthritis: The role of ultrasound in the differential diagnosis. Rheumatol. Int..

[26] Kondo Y., Kaneko Y., Takeuchi T. (2022). Differential Diagnosis of Inflammatory Arthritis from Musculoskeletal Ultrasound View. Rheumatol. Immunol. Res..

[27] Sakellariou G., Scirè C.A., Adinolfi A., Batticciotto A., Bortoluzzi A., Delle Sedie A., De Lucia O., Dejaco C., Epis O.M., Filippucci E. (2020). Differential Diagnosis of Inflammatory Arthropathies by Musculoskeletal Ultrasonography: A Systematic Literature Review. Front. Med..

[28] Zabotti A., Idolazzi L., Batticciotto A., De Lucia O., Scirè C.A., Tinazzi I., Iagnocco A. (2017). Enthesitis of the hands in psoriatic arthritis: An ultrasonographic perspective. Med. Ultrason..

[29] Razmjou A.A., Brook J., Elashoff D., Kaeley G., Choi S., Kermani T., Ranganath V.K. (2020). Ultrasound and multi-biomarker disease activity score for assessing and predicting clinical response to tofacitinib treatment in patients with rheumatoid arthritis. BMC Rheumatol..

[30] Bosch P., Husic R., Ficjan A., Gretler J., Lackner A., Graninger W.B., Duftner C., Hermann J., Dejaco C. (2019). Evaluating current definitions of low disease activity in psoriatic arthritis using ultrasound. Rheumatology.

[31] Nam S.W., Kang T. (2021). A Pragmatic Application of Ultrasonography for the Assessment of Disease Activity in Patients with Early Inflammatory Arthritis. J. Clin. Med..

[32] Pukšić S., Bolton-King P., Sexton J., Michelsen B., Kvien T.K., Hammer H.B. (2018). DAPSA and ultrasound show different perspectives of psoriatic arthritis disease activity: Results from a 12-month longitudinal observational study in patients starting treatment with biological disease-modifying antirheumatic drugs. RMD Open.

[33] Chen C.-C., Chen D.-Y. (2023). The Clinical Utility of Musculoskeletal Ultrasound for Disease Activity Evaluation and Therapeutic Response Prediction in Rheumatoid Arthritis Patients: A Narrative Review. J. Med. Ultrasound..

[34] Zhou L., Wang G., Liu X., Song J., Chen L., Xu H. (2017). Matrix metalloproteinase-3 and the 7-joint ultrasound score in the assessment of disease activity and therapeutic efficacy in patients with moderate to severe rheumatoid arthritis. Arthritis Res. Ther..

[35] Mortada M., Aly H., Elmallah R., Radwan A., Elsaman A. (2021). Construct validity and response to therapy of the U9 ultrasonographic scale for assessment of disease activity in rheumatoid arthritis. Reumatologia.

[36] Aga A.-B., Hammer H.B., Olsen I.C., Uhlig T., Kvien T.K., van der Heijde D., Fremstad H., Madland T.M., Lexberg S., Haukeland H. (2016). Development of a feasible and responsive ultrasound inflammation score for rheumatoid arthritis through a data-driven approach. RMD Open.

[37] Sapundzhieva T., Karalilova R., Batalov A. (2018). Musculoskeletal ultrasound for predicting remission in patients with rheumatoid arthritis: Results from a 1-year prospective study. Rheumatol. Int..

[38] D’agostino M.A., Schett G., López-Rdz A., Šenolt L., Fazekas K., Burgos-Vargas R., Maldonado-Cocco J., Naredo E., Carron P., Duggan A.-M. (2022). Response to secukinumab on synovitis using Power Doppler ultrasound in psoriatic arthritis: 12-week results from a phase III study, ULTIMATE. Rheumatology.

[39] Ceccarelli F., Lucchetti R., Perricone C., Spinelli F.R., Cipriano E., Truglia S., Miranda F., Riccieri V., Di Franco M., Scrivo R. (2019). Musculoskeletal ultrasound in monitoring response to apremilast in psoriatic arthritis patients: Results from a longitudinal study. Clin. Rheumatol..

[40] Agache M., Popescu C.C., Enache L., Mogoșan C., Filippucci E., Codreanu C. (2024). Additional Value of Ultrasound in Patients with Psoriatic Arthritis within Treatment Target. J. Clin. Med..

[41] Dale J., Stirling A., Zhang R., Purves D., Foley J., Sambrook M., Conaghan P.G., van der Heijde D., McConnachie A., McInnes I.B. (2016). Targeting ultrasound remission in early rheumatoid arthritis: The results of the TaSER study, a randomised clinical trial. Ann. Rheum. Dis..

[42] Haavardsholm E.A., Aga A.-B., Olsen I.C., Lillegraven S., Hammer H.B., Uhlig T., Fremstad H., Madland T.M., Lexberg S., Haukeland H. (2016). Ultrasound in management of rheumatoid arthritis: Arctic randomised controlled strategy trial. BMJ.

[43] Sapundzhieva T., Karalilova R., Batalov A. (2018). Musculoskeletal ultrasound as a biomarker of remission—Results from a one-year prospective study in patients with rheumatoid arthritis. Med. Ultrason..

[44] Smolen J.S., Landewé R.B.M., Bergstra S.A., Kerschbaumer A., Sepriano A., Aletaha D., Caporali R., Edwards C.J., Hyrich K.L., Pope J.E. (2023). EULAR recommendations for the management of rheumatoid arthritis with synthetic and biological disease-modifying antirheumatic drugs: 2022 update. Ann. Rheum. Dis..

[45] Ruyssen-Witrand A., Guernec G., Dupont J., Lapuyade D., Lioté F., Vittecoq O., Degboé Y., Constantin A. (2023). Ten-year radiographic and functional outcomes in rheumatoid arthritis patients in remission compared to patients in low disease activity. Arthritis Res. Ther..

[46] Zufferey P., Möller B., Brulhart L., Tamborrini G., Scherer A., Finckh A., Ziswiler H.-R. (2014). Persistence of ultrasound synovitis in patients with rheumatoid arthritis fulfilling the DAS28 and/or the new ACR/EULAR RA remission definitions: Results of an observational cohort study. Jt. Bone Spine.

[47] Fakhfakh R., Elamri N., Baccouche K., Laataoui S., Zeglaoui H., Bouajina E. (2021). Ultrasound remission in patients with rheumatoid arthritis in clinical remission. Reumatologia.

[48] El Miedany Y., El Gaafary M., Youssef S., Ahmed I., Bahlas S., Hegazi M., Nasr A. (2016). Optimizing therapy in inflammatory arthritis: Prediction of relapse after tapering or stopping treatment for rheumatoid arthritis patients achieving clinical and radiological remission. Clin. Rheumatol..

[49] Saleem B., Brown A.K., Quinn M., Karim Z., Hensor E.M.A., Conaghan P., Peterfy C., Wakefield R.J., Emery P. (2012). Can flare be predicted in DMARD treated RA patients in remission, and is it important? A cohort study. Ann. Rheum. Dis..

[50] Maassen J.M., van Ouwerkerk L., Allaart C.F. (2021). Tapering of disease-modifying antirheumatic drugs: An overview for daily practice. Lancet Rheumatol..

[51] Han J., Geng Y., Deng X., Zhang Z. (2016). Subclinical Synovitis Assessed by Ultrasound Predicts Flare and Progressive Bone Erosion in Rheumatoid Arthritis Patients with Clinical Remission: A Systematic Review and Metaanalysis. J. Rheumatol..

[52] David P., Di Matteo A., Hen O., Dass S., Marzo-Ortega H., Wakefield R.J., Bissell L., Nam J., Mankia K., Emery P. (2024). Poly-Refractory Rheumatoid Arthritis: An Uncommon Subset of Difficult to Treat Disease with Distinct Inflammatory and Noninflammatory Phenotypes. Arthritis Rheumatol..

[53] Ko C.-H., Chen J.-F., Cheng T.-T., Lai H.-M., Chen Y.-C. (2018). Biological tapering and sonographic flare in rheumatoid arthritis. J. Investig. Med..

[54] Sakellariou G., Conaghan P.G., Zhang W., Bijlsma J.W.J., Boyesen P., D’Agostino M.A., Doherty M., Fodor D., Kloppenburg M., Miese F. (2017). EULAR recommendations for the use of imaging in the clinical management of peripheral joint osteoarthritis. Ann. Rheum. Dis..

[55] Abicalaf C.A.R.P., Nakada L.N., dos Santos F.R.A., Akiho I., dos Santos A.C.A., Imamura M., Battistella L.R. (2021). Ultrasonography findings in knee osteoarthritis: A prospective observational cross-sectional study of 100 patients. Sci. Rep..

[56] Neogi T., Jansen T.L.T.A., Dalbeth N., Fransen J., Schumacher H.R., Berendsen D., Brown M., Choi H., Edwards N.L., Janssens H.J.E.M. (2015). 2015 Gout Classification Criteria: An American College of Rheumatology/European League Against Rheumatism collaborative initiative. Arthritis Rheumatol..

[57] Naredo E., Uson J., Jiménez-Palop M., Martínez A., Vicente E., Brito E., Rodríguez A., Cornejo F.J., Castañeda S., Martínez M.J. (2014). Ultrasound-detected musculoskeletal urate crystal deposition: Which joints and what findings should be assessed for diagnosing gout?. Ann. Rheum. Dis..

[58] Lee Y.H., Song G.G. (2018). Diagnostic accuracy of ultrasound in patients with gout: A meta-analysis. Semin. Arthritis Rheum..

[59] Hammer H.B., Karoliussen L., Terslev L., Haavardsholm E.A., Kvien T.K., Uhlig T. (2020). Ultrasound shows rapid reduction of crystal depositions during a treat-to-target approach in gout patients: 12-month results from the NOR-Gout study. Ann. Rheum. Dis..

[60] Ebstein E., Forien M., Norkuviene E., Richette P., Mouterde G., Daien C., Ea H.-K., Brière C., Lioté F., Petraitis M. (2020). UltraSound evaluation in follow-up of urate-lowering therapy in gout phase 2 (USEFUL-2): Duration of flare prophylaxis. Jt. Bone Spine.

[61] Cipolletta E., Abhishek A., Di Battista J., Grassi W., Filippucci E. (2023). Ultrasonography in the prediction of gout flares: A 12-month prospective observational study. Rheumatology.

[62] Han L., Li R., Dalbeth N., Liu M., Yu Q., Jiang C., Ning C., Liu Z., He Y., Li M. (2024). The value of musculoskeletal ultrasound in predicting gout flares in index joints: A prospective cohort study of people with gout starting urate-lowering therapy. Semin. Arthritis Rheum..

[63] Di Matteo A., Filippucci E., Salaffi F., Carotti M., Carboni D., Di Donato E., Grassi W. (2017). Diagnostic accuracy of musculoskeletal ultrasound and conventional radiography in the assessment of the wrist triangular fibrocartilage complex in patients with definite diagnosis of calcium pyrophosphate dihydrate deposition disease. Clin. Exp. Rheumatol..

[64] Forien M., Combier A., Gardette A., Palazzo E., Dieudé P., Ottaviani S. (2018). Comparison of ultrasonography and radiography of the wrist for diagnosis of calcium pyrophosphate deposition. Jt. Bone Spine.

[65] Cipolletta E., Filippou G., Scirè C., Di Matteo A., Di Battista J., Salaffi F., Grassi W., Filippucci E. (2021). The diagnostic value of conventional radiography and musculoskeletal ultrasonography in calcium pyrophosphate deposition disease: A systematic literature review and meta-analysis. Osteoarthr. Cartil..

[66] Filippou G., Scirè C.A., Damjanov N., Adinolfi A., Carrara G., Picerno V., Toscano C., Bruyn G.A., D’agostino M.A., Sedie A.D. (2017). Definition and Reliability Assessment of Elementary Ultrasonographic Findings in Calcium Pyrophosphate Deposition Disease: A Study by the OMERACT Calcium Pyrophosphate Deposition Disease Ultrasound Subtask Force. J. Rheumatol..

[67] Filippou G., Scirè C.A., Adinolfi A., Damjanov N.S., Carrara G., Bruyn G.A.W., Cazenave T., D’agostino M.A., Sedie A.D., Di Sabatino V. (2018). Identification of calcium pyrophosphate deposition disease (CPPD) by ultrasound: Reliability of the OMERACT definitions in an extended set of joints-an international multiobserver study by the OMERACT Calcium Pyrophosphate Deposition Disease Ultrasound Subtask Force. Ann. Rheum. Dis..

[68] Abhishek A., Tedeschi S.K., Pascart T., Latourte A., Dalbeth N., Neogi T., Fuller A., Rosenthal A., Becce F., Bardin T. (2023). The 2023 ACR/EULAR Classification Criteria for Calcium Pyrophosphate Deposition Disease. Arthritis Rheumatol..

[69] Sirotti S., Terslev L., Filippucci E., Iagnocco A., Moller I., Naredo E., Vreju F.A., Adinolfi A., Becce F., Hammer H.B. (2023). Development and validation of an OMERACT ultrasound scoring system for the extent of calcium pyrophosphate crystal deposition at the joint level and patient level. Lancet Rheumatol..

[70] Filippou G., Sirotti S., Cipolletta E., Filippucci E. (2024). Optimizing the Use of Ultrasound in Calcium Pyrophosphate Deposition (CPPD): A Review from the Ground Up. Gout Urate Cryst. Depos. Dis..

[71] Hughes M., Bruni C., Cuomo G., Sedie A.D., Gargani L., Gutierrez M., Lepri G., Ruaro B., Santiago T., Suliman Y. (2021). The role of ultrasound in systemic sclerosis: On the cutting edge to foster clinical and research advancement. J. Scleroderma Relat. Disord..

[72] Naredo E., Pascau J., Damjanov N., Lepri G., Gordaliza P.M., Janta I., Ovalles-Bonilla J.G., López-Longo F.J., Matucci-Cerinic M. (2020). Performance of ultra-high-frequency ultrasound in the evaluation of skin involvement in systemic sclerosis: A preliminary report. Rheumatology.

[73] Santiago T., Santos E., Ruaro B., Lepri G., Green L., Wildt M., Watanabe S., Lescoat A., Hesselstrand R., Del Galdo F. (2022). Ultrasound and elastography in the assessment of skin involvement in systemic sclerosis: A systematic literature review focusing on validation and standardization—WSF Skin Ultrasound Group. Semin. Arthritis Rheum..

[74] Santiago T., Santos E.J.F., Ruaro B., Lepri G., Green L., Wildt M., Watanabe S., Lescoat A., Hesselstrand R., del Galdo F. (2022). Recommendations for the execution and reporting of skin ultrasound in systemic sclerosis: An international collaboration under the WSF skin ultrasound group. RMD Open.

[75] Suliman Y.A., Bruni C., Hughes M., Matucci-Cerinic M., Furst D.E. (2021). Ultrasonographic imaging of systemic sclerosis digital ulcers: A systematic literature review and validation steps. Semin. Arthritis Rheum..

[76] Sandler R.D., Matucci-Cerinic M., Hughes M. (2020). Musculoskeletal hand involvement in systemic sclerosis. Semin. Arthritis Rheum..

[77] Balbach M.L., Corty R., Hill B., Frech T., Aslam F., Chew E.Y. (2024). Development of a Musculoskeletal Ultrasound Protocol to Evaluate Hand Pain in Systemic Sclerosis Patients. Diagnostics.

[78] Avouac J., Walker U.A., Hachulla E., Riemekasten G., Cuomo G., Carreira P.E., Caramaschi P., Ananieva L.P., Matucci-Cerinic M., Czirjak L. (2016). Joint and tendon involvement predict disease progression in systemic sclerosis: A EUSTAR prospective study. Ann. Rheum. Dis..

[79] Volkmann E.R., Andréasson K., Smith V. (2023). Systemic sclerosis. Lancet.

[80] Khanna D., Distler O., Cottin V., Brown K.K., Chung L., Goldin J.G., Matteson E.L., Kazerooni E.A., Walsh S.L., McNitt-Gray M. (2022). Diagnosis and monitoring of systemic sclerosis-associated interstitial lung disease using high-resolution computed tomography. J. Scleroderma Relat. Disord..

[81] Guerra M.G., Pinto T.M., Águeda A., Rodrigues J., Marona J., Violante A., Oliveira M. (2023). The Role of Lung Ultrasound in Systemic Sclerosis: A Systematic Review. J. Clin. Rheumatol..

[82] Beigi D.M.R., Pellegrino G., Loconte M., Landini N., Mattone M., Paone G., Truglia S., Di Ciommo F.R., Bisconti I., Cadar M. (2024). Lung ultrasound compared to computed tomography detection and automated quantification of systemic sclerosis-associated interstitial lung disease: Preliminary study. Rheumatology.

[83] Fairchild R., Chung M., Yang D., Sharpless L., Li S., Chung L. (2021). Development and Assessment of Novel Lung Ultrasound Interpretation Criteria for the Detection of Interstitial Lung Disease in Systemic Sclerosis. Arthritis Care Res..

[84] Gargani L., Bruni C., Romei C., Frumento P., Moreo A., Agoston G., Guiducci S., Bellando-Randone S., Lepri G., Belloli L. (2020). Prognostic Value of Lung Ultrasound B-Lines in Systemic Sclerosis. Chest.

[85] Hassan R.I., Lubertino L.I., Barth M.A., Quaglia M.F., Montoya S.F., Kerzberg E., Binda M.d.C. (2019). Lung Ultrasound as a Screening Method for Interstitial Lung Disease in Patients With Systemic Sclerosis. J. Clin. Rheumatol..

[86] Tardella M., Di Carlo M., Carotti M., Filippucci E., Grassi W., Salaffi F. (2018). Ultrasound B-lines in the evaluation of interstitial lung disease in patients with systemic sclerosis: Cut-off point definition for the presence of significant pulmonary fibrosis. Medicine.

[87] Barskova T., Gargani L., Guiducci S., Randone S.B., Bruni C., Carnesecchi G., Conforti M.L., Porta F., Pignone A., Caramella D. (2013). Lung ultrasound for the screening of interstitial lung disease in very early systemic sclerosis. Ann. Rheum. Dis..

[88] Radić M., Đogaš H., Gelemanović A., Petričević S.J., Škopljanac I., Radić J. (2023). Pulmonary Ultrasonography in Systemic Sclerosis-Induced Interstitial Lung Disease-A Systematic Review and Meta-Analysis. Diagnostics.

[89] Xie H.Q., Zhang W.W., Sun D.S., Chen X.M., Yuan S.F., Gong Z.H., Liu L. (2019). A simplified lung ultrasound for the diagnosis of interstitial lung disease in connective tissue disease: A meta-analysis. Arthritis Res. Ther..

[90] Shumilova A., Vital E.M. (2023). Musculoskeletal manifestations of systemic lupus erythematosus. Best Pract. Res. Clin. Rheumatol..

[91] Ohmura K. (2021). Which is the best SLE activity index for clinical trials?. Mod. Rheumatol..

[92] Zayat A.S., Yusof Y.M., Wakefield R.J., Conaghan P.G., Emery P., Vital E.M. (2016). The role of ultrasound in assessing musculoskeletal symptoms of systemic lupus erythematosus: A systematic literature review. Rheumatology.

[93] Zayat A.S., Mahmoud K., Yusof Y.M., Mukherjee S., D’agostino M.-A., Hensor E.M.A., Wakefield R.J., Conaghan P.G., Edwards C.J., Emery P. (2019). Defining inflammatory musculoskeletal manifestations in systemic lupus erythematosus. Rheumatology.

[94] Di Matteo A., Filippucci E., Cipolletta E., Satulu I., Hurnakova J., Lato V., De Angelis R., Horvath R., Pavelka K., Salaffi F. (2018). Entheseal involvement in patients with systemic lupus erythematosus: An ultrasound study. Rheumatology.

[95] Fagni F., Bettiol A., Silvestri E., Fedi R., Palermo A., Urban M.L., Mazzotta R., Malandrino D., Bello F., Mattioli I. (2023). Prevalence and clinical associations of ultrasound-confirmed enthesitis in systemic lupus erythematosus. Rheumatology.

[96] Emerah A., Mostafa S., Kotb L., Amer Y., Ismail B., Sarhan S.A. (2024). The burden of entheseal involvement in systemic lupus erythematosus: A comparative ultrasonograghic study. Clin. Rheumatol..

[97] Mahmoud K., Zayat A.S., Yusof Y.M., Dutton K., Teh L.S., Yee C.-S., D’cruz D., Ng N., Isenberg D., Ciurtin C. (2021). Ultrasound to identify systemic lupus erythematosus patients with musculoskeletal symptoms who respond best to therapy: The US Evaluation For mUsculoskeletal Lupus longitudinal multicentre study. Rheumatology.

[98] Habers G.E.A., Van Brussel M., Bhansing K.J., Hoppenreijs E.P., Janssen A.J., Van Royen-Kerkhof A., Pillen S. (2015). Quantitative muscle ultrasonography in the follow-up of juvenile dermatomyositis. Muscle Nerve.

[99] Di Matteo A., Moscioni E., Lommano M.G., Cipolletta E., Smerilli G., Farah S., Airoldi C., Aydin S.Z., Becciolini A., Bonfiglioli K. (2023). Reliability assessment of ultrasound muscle echogenicity in patients with rheumatic diseases: Results of a multicenter international web-based study. Front. Med..

[100] Paramalingam S., Morgan K., Becce F., Diederichsen L.P., Ikeda K., Mandl P., Ohrndorf S., Sedie A.D., Sharp V., Tan A.L. (2021). Conventional ultrasound and elastography as imaging outcome tools in autoimmune myositis: A systematic review by the OMERACT ultrasound group. Semin. Arthritis Rheum..

[101] Alfuraih A.M., O’connor P., Tan A.L., Hensor E.M.A., Ladas A., Emery P., Wakefield R.J. (2019). Muscle shear wave elastography in idiopathic inflammatory myopathies: A case-control study with MRI correlation. Skelet. Radiol..

[102] Bachasson D., Dubois G.J., Allenbach Y., Benveniste O., Hogrel J.-Y. (2018). Muscle Shear Wave Elastography in Inclusion Body Myositis: Feasibility, Reliability and Relationships with Muscle Impairments. Ultrasound Med. Biol..

[103] Song Y., Lee S., Yoo D.H., Jang K.-S., Bae J. (2016). Strain sonoelastography of inflammatory myopathies: Comparison with clinical examination, magnetic resonance imaging and pathologic findings. Br. J. Radiol..

[104] Tan J.Y., Tan C.Y., Yahya M.A., Shahrizaila N., Goh K.J. (2024). Evaluating disease status in idiopathic inflammatory myopathies with quantitative muscle ultrasound. Muscle Nerve.

[105] Walter A.W., Lim J., Raaphorst J., Smithuis F.F., Harder J.M.D., Eftimov F., Potters W., Saris C.G.J., de Visser M., van Schaik I.N. (2022). Ultrasound and MR muscle imaging in new onset idiopathic inflammatory myopathies at diagnosis and after treatment: A comparative pilot study. Rheumatology.

[106] Zhao R., Li X., Jiang Y., Su N., Li J., Kang L., Zhang Y., Yang M. (2022). Evaluation of Appendicular Muscle Mass in Sarcopenia in Older Adults Using Ultrasonography: A Systematic Review and Meta-Analysis. Gerontology.

[107] Kara M., Kaymak B., Frontera W., Ata A., Ricci V., Ekiz T., Chang K., Han D., Michail X., Quittan M. (2021). Diagnosing sarcopenia: Functional perspectives and a new algorithm from the ISarcoPRM. J. Rehabil. Med..

[108] Casey P., Alasmar M., McLaughlin J., Ang Y., McPhee J., Heire P., Sultan J. (2022). The current use of ultrasound to measure skeletal muscle and its ability to predict clinical outcomes: A systematic review. J. Cachexia Sarcopenia Muscle.

[109] Mah J.K., van Alfen N. (2018). Neuromuscular Ultrasound: Clinical Applications and Diagnostic Values. Can. J. Neurol. Sci..

[110] Hwang H.-E., Hsu T.-R., Lee Y.-H., Wang H.-K., Chiou H.-J., Niu D.-M. (2017). Muscle ultrasound: A useful tool in newborn screening for infantile onset pompe disease. Medicine.

[111] Jansen M., van Alfen N., van der Sanden M.W.N., van Dijk J.P., Pillen S., de Groot I.J. (2012). Quantitative muscle ultrasound is a promising longitudinal follow-up tool in Duchenne muscular dystrophy. Neuromuscul. Disord..

[112] Tsuji Y., Noto Y.-I., Shiga K., Teramukai S., Nakagawa M., Mizuno T. (2017). A muscle ultrasound score in the diagnosis of amyotrophic lateral sclerosis. Clin. Neurophysiol..

[113] Tan A.L., Di Matteo A., Wakefield R.J., Biglands J. (2023). Update on muscle imaging in myositis. Curr. Opin. Rheumatol..

[114] Lorenzon M., Spina E., Di Franco F.T., Giovannini I., De Vita S., Zabotti A. (2022). Salivary Gland Ultrasound in Primary Sjögren’s Syndrome: Current and Future Perspectives. Open Access Rheumatol..

[115] Zabotti A., Callegher S.Z., Tullio A., Vukicevic A., Hocevar A., Milic V., Cafaro G., Carotti M., Delli K., De Lucia O. (2020). Salivary Gland Ultrasonography in Sjögren’s Syndrome: A European Multicenter Reliability Exercise for the HarmonicSS Project. Front. Med..

[116] Jousse-Joulin S., D’Agostino M.A., Nicolas C., Naredo E., Ohrndorf S., Backhaus M., Tamborrini G., Chary-Valckenaere I., Terslev L., Iagnocco A. (2019). Video clip assessment of a salivary gland ultrasound scoring system in Sjögren’s syndrome using consensual definitions: An OMERACT ultrasound working group reliability exercise. Ann. Rheum. Dis..

[117] Takagi Y., Nakamura H., Sumi M., Shimizu T., Hirai Y., Horai Y., Takatani A., Kawakami A., Eida S., Sasaki M. (2018). Combined classification system based on ACR/EULAR and ultrasonographic scores for improving the diagnosis of Sjögren’s syndrome. PLoS ONE.

[118] van Nimwegen J.F., Mossel E., Delli K., van Ginkel M.S., Stel A.J., Kroese F.G.M., Spijkervet F.K.L., Vissink A., Arends S., Bootsma H. (2020). Incorporation of Salivary Gland Ultrasonography Into the American College of Rheumatology/European League Against Rheumatism Criteria for Primary Sjögren’s Syndrome. Arthritis Care Res..

[119] La Paglia G.M.C., Sanchez-Pernaute O., Alunno A., Martínez-Becerra M.J., Romero-Bueno F., Recuero S., Borges P.E., Mahillo-Fernández I., Garrido J., Gerli R. (2020). Ultrasound salivary gland involvement in Sjogren’s syndrome vs. other connective tissue diseases: Is it autoantibody and gland dependent?. Clin. Rheumatol..

[120] Law S.T., Jafarzadeh S.R., Govender P., Sun X., Sanchorawala V., Kissin E.Y. (2020). Comparison of ultrasound features of major salivary glands in sarcoidosis, amyloidosis, and Sjögren’s syndrome. Arthritis Care Res..

[121] Sebastian A., Madej M., Sebastian M., Butrym A., Woytala P., Hałoń A., Wiland P. (2020). Prevalence and clinical presentation of lymphoproliferative disorder in patients with primary Sjögren’s syndrome. Rheumatol. Int..

[122] Geng Z., Ye C., Zhu X. (2023). Malignancies in systemic rheumatic diseases: A mini review. Front. Immunol..

[123] Lorenzon M., Di Franco F.T., Zabotti A., Pegolo E., Giovannini I., Manfrè V., Mansutti E., De Vita S., Zuiani C., Girometti R. (2021). Sonographic features of lymphoma of the major salivary glands diagnosed with ultrasound-guided core needle biopsy in Sjögren’s syndrome. Clin. Exp. Rheumatol..

[124] Khalife A., Bakhshaee M., Davachi B., Mashhadi L., Khazaeni K. (2016). The diagnostic value of B-mode sonography in differentiation of malignant and benign tumors of the parotid gland. Iran. J. Otorhinolaryngol..

[125] Zabotti A., Callegher S.Z., Lorenzon M., Pegolo E., Scott C.A., Tel A., Giovannini I., Robiony M., Di Loreto C., Zuiani C. (2021). Ultrasound-guided core needle biopsy compared with open biopsy: A new diagnostic approach to salivary gland enlargement in Sjögren’s syndrome?. Rheumatology.

[126] Baer A.N., Grader-Beck T., Antiochos B., Birnbaum J., Fradin J.M. (2021). Ultrasound-guided biopsy of suspected salivary gland lymphoma in Sjögren’s syndrome. Arthritis Care Res..

[127] Diekhoff T., Fischer T., Schefer Q., Posch M.G., Dörner T., Laurent D., Li Y., Wagner F.D., Oliver S.J. (2020). Ianalumab (VAY736) in primary Sjögren’s syndrome: Assessing disease activity using multi-modal ultrasound. Clin. Exp. Rheumatol..

[128] Fisher B.A., Everett C.C., Rout J., O’dwyer J.L., Emery P., Pitzalis C., Ng W.-F., Carr A., Pease C.T., Price E.J. (2018). Effect of rituximab on a salivary gland ultrasound score in primary Sjögren’s syndrome: Results of the TRACTISS randomised double-blind multicentre substudy. Ann. Rheum. Dis..

[129] Oruk Y.E., Çildağ M.B., Karaman C.Z., Çildağ S. (2021). Effectiveness of ultrasonography and shear wave sonoelastography in Sjögren syndrome with salivary gland involvement. Ultrasonography.

[130] Turnaoglu H., Rahatli F.K., Pamukcu M., Haberal K.M., Uslu N. (2018). Diagnostic value of acustic radiation force impulse imaging in the assessment of salivary gland involvement in primary Sjögren’s sydrome. Med. Ultrason..

[131] Bădărînză M., Serban O., Maghear L., Bocsa C., Micu M., Damian L., Felea I., Fodor D. (2020). Shear wave elastography as a new method to identify parotid lymphoma in primary Sjögren Syndrome patients: An observational study. Rheumatol. Int..

[132] Dasgupta B., Cimmino M.A., Maradit-Kremers H., Schmidt W.A., Schirmer M., Salvarani C., Bachta A., Dejaco C., Duftner C., Jensen H.S. (2012). 2012 provisional classification criteria for polymyalgia rheumatica: A European League Against Rheumatism/American College of Rheumatology collaborative initiative. Ann. Rheum. Dis..

[133] Mackie S.L., Koduri G., Hill C.L., Wakefield R.J., Hutchings A., Loy C., Dasgupta B., Wyatt J.C. (2015). Accuracy of musculoskeletal imaging for the diagnosis of polymyalgia rheumatica: Systematic review. RMD Open.

[134] Todorov P.T., Batalov A.Z. (2023). Ultrasound description and follow up of painful cervical interspinous bursitis in a Polymyalgia Rheumatica patient—A case report. Med. Ultrason..

[135] Sagawa R., Kida T., Inoue H., Hirano A., Kohno M., Kawahito Y. (2022). Lumbar interspinous bursitis in a patient with polymyalgia rheumatica/giant cell arteritis detected by musculoskeletal ultrasound: A case report. RMD Open.

[136] Conticini E., Falsetti P., D’alessandro M., Al Khayyat S.G., Grazzini S., Baldi C., Acciai C., Gentileschi S., D’alessandro R., Bellisai F. (2023). Clinical, laboratory and ultrasonographic findings at baseline predict long-term outcome of polymyalgia rheumatica: A multicentric retrospective study : Polymyalgia rheumatica predicted by ultrasonographic findings polymyalgia rheumatica outcome predicted early by ultrasound. Intern. Emerg. Med..

[137] Dejaco C., Singh Y.P., Perel P., Hutchings A., Camellino D., Mackie S., Abril A., Bachta A., Balint P., Barraclough K. (2015). European League Against Rheumatism; American College of Rheumatology. 2015 recommendations for the management of polymyalgia rheumatica: A European League against Rheumatism/American College of Rheumatology collaborative initiative. Arthritis Rheumatol..

[138] Figus F.A., Skoczyńska M., McConnell R., Massazza G., Iagnocco A. (2021). Imaging in polymyalgia rheumatica: Which technique to use?. Clin. Exp. Rheumatol..

[139] Suzuki T., Yoshida R., Hidaka Y., Seri Y. (2017). Proliferative Synovitis of the Shoulder Bursae is a Key Feature for Discriminating Elderly Onset Rheumatoid Arthritis Mimicking Polymyalgia Rheumatica from Polymyalgia Rheumatica. Clin. Med. Insights Arthritis Musculoskelet. Disord..

[140] Ottaviani S., Goossens J., Demaria L., Forien M., Palazzo E., Dieudé P. (2020). Ultrasound shoulder assessment of calcium pyrophosphate disease with suspected polymyalgia rheumatica. Clin. Exp. Rheumatol..

[141] Schmidt W.A., Schäfer V.S. (2024). Diagnosing vasculitis with ultrasound: Findings and pitfalls. Ther. Adv. Musculoskelet. Dis..

[142] Grayson P.C., Ponte C., Suppiah R., Robson J.C., Gribbons K.B., Judge A., Craven A., Khalid S., Hutchings A., Danda D. (2022). 2022 American College of Rheumatology/EULAR classification criteria for Takayasu arteritis. Ann. Rheum. Dis..

[143] Ponte C., Grayson P.C., Robson J.C., Suppiah R., Gribbons K.B., Judge A., Craven A., Khalid S., Hutchings A., Watts R.A. (2022). 2022 American College of Rheumatology/EULAR Classification Criteria for Giant Cell Arteritis. Arthritis Rheumatol..

[144] Dejaco C., Ramiro S., Bond M., Bosch P., Ponte C., Mackie S.L., Bley T.A., Blockmans D., Brolin S., Bolek E.C. (2024). EULAR recommendations for the use of imaging in large vessel vasculitis in clinical practice: 2023 update. Ann. Rheum. Dis..

[145] De Miguel E., Macchioni P., Conticini E., Campochiaro C., Karalilova R., Monti S., Ponte C., Klinowski G., Monjo-Henry I., Falsetti P. (2024). Prevalence and characteristics of subclinical giant cell arteritis in polymyalgia rheumatica. Rheumatology.

[146] Schmidt W.A. (2023). Monitoring giant cell arteritis with ultrasound. Rheumatology.

[147] Cowley S., Harkins P., Kirby C., Conway R., Kane D.J. (2024). Should all patients with polymyalgia rheumatica have a vascular ultrasound assessment?. Ann. Rheum. Dis..

[148] Terslev L., Naredo E., Keen H.I., Bruyn G.A., Iagnocco A., Wakefield R.J., Conaghan P.G., Maxwell L.J., Beaton D.E., Boers M. (2019). The OMERACT Stepwise Approach to Select and Develop Imaging Outcome Measurement Instruments: The Musculoskeletal Ultrasound Example. J. Rheumatol..

[149] Nielsen B.D., Therkildsen P., Keller K.K., Gormsen L.C., Hansen I.T., Hauge E.-M. (2023). Ultrasonography in the assessment of disease activity in cranial and large-vessel giant cell arteritis: A prospective follow-up study. Rheumatology.

[150] Lawande A.D., Warrier S.S., Joshi M.S. (2014). Role of ultrasound in evaluation of peripheral nerves. Indian J. Radiol. Imaging.

[151] Schwabl C., Schmidle G., Kaiser P., Drakonaki E., Taljanovic M.S., Klauser A.S. (2023). Nerve entrapment syndromes: Detection by ultrasound. Ultrasonography.

[152] Martinoli C., Bianchi S., Gandolfo N., Valle M., Simonetti S., Derchi L.E. (2000). US of nerve entrapments in osteofibrous tunnels of the upper and lower limbs. Radiographics.

[153] Shaukat A., Aamir H., Ahmad Z., Ahmad U. (2024). Diagnostic accuracy of ultrasonography in diagnosis of Carpal Tunnel Syndrome. Pak. J. Med. Sci..

[154] Ratasvuori M., Sormaala M., Kinnunen A., Lindfors N. (2022). Ultrasonography for the diagnosis of carpal tunnel syndrome: Correlation of clinical symptoms, cross-sectional areas and electroneuromyography. J. Hand Surg. Eur. Vol..

[155] Huang Z., Du S., Qi Y., Chen G., Yan W. (2015). Effectiveness of Ultrasound Guidance on Intraarticular and Periarticular Joint Injections: Systematic Review and Meta-analysis of Randomized Trials. Am. J. Phys. Med. Rehabil..

[156] Uson J., Rodriguez-García S.C., Castellanos-Moreira R., O’Neill T.W., Doherty M., Boesen M., Pandit H., Parera I.M., Vardanyan V., Terslev L. (2021). EULAR recommendations for intra-articular therapies. Ann. Rheum. Dis..

[157] Sconfienza L.M., Adriaensen M., Albano D., Allen G., Gómez M.P.A., Bazzocchi A., Beggs I., Bignotti B., Chianca V., Corazza A. (2020). Clinical indications for image-guided interventional procedures in the musculoskeletal system: A Delphi-based consensus paper from the European Society of Musculoskeletal Radiology (ESSR)-part I, shoulder. Eur. Radiol..

[158] Sconfienza L.M., Adriaensen M., Albano D., Gómez M.P.A., Bazzocchi A., Beggs I., Bignotti B., Chianca V., Corazza A., Dalili D. (2020). Ultrasound and Interventional Subcommittees of the European Society of Musculoskeletal Radiology (ESSR). Clinical indications for image-guided interventional procedures in the musculoskeletal system: A Delphi-based consensus paper from the European Society of Musculoskeletal Radiology (ESSR)-Part II, elbow and wrist. Eur. Radiol..

[159] Sconfienza L.M., Adriaensen M., Albano D., Allen G., Gómez M.P.A., Bazzocchi A., Beggs I., Bignotti B., Chianca V., Corazza A. (2020). Ultrasound and Interventional Subcommittees of the European Society of Musculoskeletal Radiology (ESSR). Clinical indications for image guided interventional procedures in the musculoskeletal system: A Delphi-based consensus paper from the European Society of Musculoskeletal Radiology (ESSR)-part III, nerves of the upper limb. Eur. Radiol..

[160] Sconfienza L.M., Adriaensen M., Alcala-Galiano A., Allen G., Gómez M.P.A., Aringhieri G., Bazzocchi A., Beggs I., Chianca V., Corazza A. (2022). Clinical indications for image-guided interventional procedures in the musculoskeletal system: A Delphi-based consensus paper from the European Society of Musculoskeletal Radiology (ESSR)-part IV, hip. Eur. Radiol..

[161] Sconfienza L.M., Adriaensen M., Albano D., Alcala-Galiano A., Allen G., Gómez M.P.A., Aringhieri G., Bazzocchi A., Beggs I., Chianca V. (2022). Clinical indications for image-guided interventional procedures in the musculoskeletal system: A Delphi-based consensus paper from the European Society of Musculoskeletal Radiology (ESSR)-part V, knee. Eur. Radiol..

[162] Sconfienza L.M., Adriaensen M., Albano D., Alcala-Galiano A., Allen G., Gómez M.P.A., Aringhieri G., Bazzocchi A., Beggs I., Chianca V. (2022). Clinical indications for image-guided interventional procedures in the musculoskeletal system: A Delphi-based consensus paper from the European Society of Musculoskeletal Radiology (ESSR)-part VI, foot and ankle. Eur. Radiol..

[163] Aly A.-R., Rajasekaran S., Ashworth N. (2015). Ultrasound-guided shoulder girdle injections are more accurate and more effective than landmark-guided injections: A systematic review and meta-analysis. Br. J. Sports Med..

[164] Lyubomir S., Tanya S., Anastas B., Marya S., Sevdalina L. (2020). Study on Accuracy of Blind and Ultrasound-Guided Arthrocentesis of Hip Joint. J. Rheum. Dis. Treat..

[165] Fang W.H., Chen X.T., Vangsness C.T. (2021). Ultrasound-Guided Knee Injections Are More Accurate Than Blind Injections: A Systematic Review of Randomized Controlled Trials. Arthrosc. Sports Med. Rehabil..

[166] Bosch P., Carubbi F., Scirè C.A., Baraliakos X., Falzon L., Dejaco C., Machado P.M. (2021). Value of imaging to guide interventional procedures in rheumatic and musculoskeletal diseases: A systematic literature review informing EULAR points to consider. RMD Open.

[167] Zadro J., Rischin A., Johnston R.V., Buchbinder R. (2021). Image-guided glucocorticoid injection versus injection without image guidance for shoulder pain. Cochrane Database Syst. Rev..

[168] Koutsianas C., Klocke R. (2017). 265. Efficacy of ultrasound-guided versus landmark-guided injections in rheumatology: A systematic review. Rheumatology.

[169] Sconfienza L.M., Adriaensen M., Albano D., Alcala-Galiano A., Allen G., Gómez M.P.A., Aringhieri G., Bazzocchi A., Beggs I., Chianca V. (2022). Clinical indications for image-guided interventional procedures in the musculoskeletal system: A Delphi-based consensus paper from the European Society of Musculoskeletal Radiology (ESSR)-part VII, nerves of the lower limb. Eur. Radiol..

[170] Fukui S., Rokutanda R., Kawaai S., Suda M., Iwata F., Okada M., Kishimoto M. (2023). Current evidence and practical knowledge for ultrasound-guided procedures in rheumatology: Joint aspiration, injection, and other applications. Best Pract. Res. Clin. Rheumatol..

[171] Mielnik P., Fojcik M., Segen J., Kulbacki M. (2018). A Novel Method of Synovitis Stratification in Ultrasound Using Machine Learning Algorithms: Results From Clinical Validation of the MEDUSA Project. Ultrasound Med. Biol..

[172] Hemalatha R., Vijaybaskar V., Thamizhvani T. (2019). Automatic localization of anatomical regions in medical ultrasound images of rheumatoid arthritis using deep learning. Proc. Inst. Mech. Eng. H.

[173] Andersen J.K.H., Pedersen J.S., Laursen M.S., Holtz K., Grauslund J., Savarimuthu T.R., Just S.A. (2019). Neural networks for automatic scoring of arthritis disease activity on ultrasound images. RMD Open.

[174] Smistad E., Johansen K.F., Iversen D.H., Reinertsen I. (2018). Highlighting nerves and blood vessels for ultrasound-guided axillary nerve block procedures using neural networks. J. Med. Imaging.

[175] Huang C., Zhou Y., Tan W., Qiu Z., Zhou H., Song Y., Zhao Y., Gao S. (2019). Applying deep learning in recognizing the femoral nerve block region on ultrasound images. Ann. Transl. Med..

[176] Faisal A., Ng S.-C., Goh S.-L., Lai K.W. (2018). Knee cartilage segmentation and thickness computation from ultrasound images. Med. Biol. Eng. Comput..

[177] Shin Y., Yang J., Lee Y.H., Kim S. (2021). Artificial intelligence in musculoskeletal ultrasound imaging. Ultrasonography.

